# A web tool for the design and management of panels of genes for targeted enrichment and massive sequencing for clinical applications

**DOI:** 10.1093/nar/gku472

**Published:** 2014-05-26

**Authors:** Alejandro Alemán, Francisco Garcia-Garcia, Ignacio Medina, Joaquín Dopazo

**Affiliations:** 1 Computational Genomics Department, Centro de Investigación Príncipe Felipe (CIPF), Valencia, 46012, Spain; 2 Bioinformatics of Rare Diseases (BIER), CIBER de Enfermedades Raras (CIBERER), Valencia, 46012, Spain; 3 Functional Genomics Node, (INB) at CIPF, Valencia, 46012, Spain

## Abstract

Disease targeted sequencing is gaining importance as a powerful and cost-effective application of high throughput sequencing technologies to the diagnosis. However, the lack of proper tools to process the data hinders its extensive adoption. Here we present TEAM, an intuitive and easy-to-use web tool that fills the gap between the predicted mutations and the final diagnostic in targeted enrichment sequencing analysis. The tool searches for known diagnostic mutations, corresponding to a disease panel, among the predicted patient's variants. Diagnostic variants for the disease are taken from four databases of disease-related variants (HGMD-public, HUMSAVAR, ClinVar and COSMIC.) If no primary diagnostic variant is found, then a list of secondary findings that can help to establish a diagnostic is produced. TEAM also provides with an interface for the definition of and customization of panels, by means of which, genes and mutations can be added or discarded to adjust panel definitions. TEAM is freely available at: http://team.babelomics.org.

## INTRODUCTION

Precision medicine relies on the transition from conventional to molecular biomarker-based diagnostics for treatment decisions. The recent development of the new generation of sequencing technologies makes rapid and economical genome sequencing possible and, consequently accelerates the ratio of biomarker discovery. However, although exome- and genome-sequencing are increasingly affordable for research, disease-targeted testing presents a number of advantages that makes it an invaluable tool in the diagnostic evaluation (1). Actually, the American College of Medical Genetics and Genomics recommends that exome or genome sequencing approaches should only be applied to those cases in which disease-targeted testing is unlikely to return a positive result in a timely and cost-effective manner or is directly negative. Along these lines, a few months ago the first Food and Drug Administration (FDA) authorization for next-generation sequencer for clinical applications was granted (2). Looking for known diagnostic variants in known disease genes optimizes the resources in a clinical context which drastically reduces the risk of occurrence of unsolicited, incidental findings (3), especially when these are not actionable (which is often the case) (4). Multiplexing assays with panels of disease genes allows high precision diagnostic of cancer subtypes or hereditary diseases at quite affordable prices.

To our knowledge, despite the increasing use of this type of diagnostic tools (see http://www.genetests.org/), there are no open bioinformatic tools available to deal with the results of such tests (beyond the conventional, general-purpose commercial software provided by the instrument manufacturers). Here we present Targeted Enrichment Analysis and Management (TEAM), a web-based solution for the definition, analysis and management of panels of genes for targeted enrichment sequencing for diagnostic purposes. TEAM allows users to define their own panels of genes and use them for diagnostic in an intuitive and easy-to-use environment. Moreover, despite TEAM being a web application, the entire patient's sequencing information is managed locally thus avoiding any problem of data privacy or confidentiality.

## METHODS

### TEAM diagnostic framework

The rationale of TEAM is to provide users with a tool that facilitates all the steps that lead to the detection of the diagnostic variant from the panel-based targeted enriched sequencing primary data. Nowadays, such steps are carried out by means of a series of unconnected tools or by general-purpose tools. A diagnostic panel for targeted enrichment sequencing can be defined as a collection of genomic regions (usually coding genes) in which some (usually known) mutations, which are diagnostic of a particular disease, syndrome or phenotype, are sought. The input data consist of patient's genomic variants predicted in these genomic regions. Such predictions are stored in files with the standard Variant Calling Format (VCF) (5). These files are generated by the software supplied with the sequencing instrument or by other similar software for primary data processing. TEAM queries several disease-related mutation databases to identify known diagnostic mutations (for the disease defined in the panel) among the variants found in the patient's sequence. If no known variants are found then TEAM provides a list of variants with a potential deleterious effect that could eventually be related to the disease. TEAM not only facilitates the process of identification of diagnostic mutations but also enables the definition of panels by means of an intuitive interface.

### Using TEAM

#### Input

Once the sequencing process is finished and quality control has been satisfactory, the instrument software (or any other primary processing software) is used for mapping reads and calling the variants. The result of this process is a VCF file containing all the variants different from the reference genome in the sample sequenced. This file can be uploaded into TEAM by using the ***VCF file*** button. It is important to note here that the entire management of the VCF file is local: no patient's sequence data is sent over the internet thus avoiding any problem of data privacy or confidentiality.

#### Use for diagnostic

Once the file has been uploaded, a panel must be chosen from the ***Panel*** list. Then, pressing the *Run* button the diagnostic process starts. TEAM searches first for known diagnostic mutation(s) taken from four databases: HGMD-public (6), HUMSAVAR (http://www.uniprot.org/docs/humsavar), ClinVar (7) and COSMIC (8). If a hit is found, a diagnostic variant is reported. In the case of a negative result, TEAM reports all the variants of uncertain effect, with possible deleterious or pathologic consequences found in the genes of the panel. The program uses the conventional PolyPhen (9) and SIFT (10) indexes, taken from the Variant program (11), to set pathogenicity thresholds (in the ***Panel Manager***, see below), which can be modified by the user if needed. Single Nucleotide Variants (SNVs) over the threshold defined (no threshold by default) and indels will be reported. It is worth noticing that the program relies on the quality of the VCF read and, it is well known that indels sometimes undergo problems of mapping.

The results are presented in two informative tabs. The first one, the ***Diagnostic*** tab, contains information on known diagnostic mutations found among the variants within the genomic regions delimited by the panel (genes of the panel). The following information is listed for any diagnostic mutation: chromosome, position, single nucleotide polymorphism (SNP) identifier (if exists), reference allele, alternative allele, affected gene name, consequence type, disease or phenotype, source (public database) in which the annotation was found, SIFT and PolyPhen pathogenicity scores (when available) as well as the PhastCons conservation score (12). Other interesting values such as Quality, OMIM, Description, etc. are not displayed by default but can also be listed by reconfiguring the view of the panel. Columns can be rearranged by dragging and dropping them. The columns to be displayed can be reconfigured by clicking on any of them, then clicking on the ***Columns*** item of the menu and selecting the desired choice. The ***Secondary findings*** tab displays the rest of the variants with deleterious potential found within the genomic regions that define the panel. The same information as in the previous tab is displayed here. In case of unsuccessful primary diagnostic, the information contained in this tab can help to suggest another causal variant (that eventually can become a new diagnostic mutation upon further validation).

#### Report

Finally, a report of the findings can be obtained by clicking the ***Generate Report*** button in the lower right corner of the tab. Then, an editable template pops up, that contains: relevant data regarding the analysis (title, date, name of the person who reports the analysis), a text box for information on the analysis, the primary diagnostic (if any), the secondary findings, a description of the panel used (mutations and genes) and another extra text box for including any comment. When the content of the template is confirmed, a formatted web page is produced, that can be printed or saved as PDF.

### Panel management

#### The panel environment

The ***Show Panels*** button displays the ***Panels*** window. This window contains the panels defined by the user plus some example panels. Any panel definition has two icons associated. One of them allows removing the panel and the other one enables panel edition. Clicking the panel edition button invokes the ***Panel Manager***, which is an interactive and intuitive panel edition that is commented in detail below. The ***Panels*** window menu offers options to: (i) create a new panel (through the ***Panel Manager***), (ii) import panel definitions previously saved in the local disk, (iii) save panel definitions in the local disk and (iv) reset all the panel definitions.

#### Concepts for panel definition

Probably, the most original and powerful option of TEAM is the possibility of defining panels in a simple and intuitive manner. There are three key concepts in the definition of a panel: regions to be captured (here genes), diagnostic mutations and phenotypes (or disease definitions). Phenotypes or disease terms are taken from four databases: HGMD-public (6), HUMSAVAR (http://www.uniprot.org/docs/humsavar), ClinVar (7) and COSMIC (8). Any disease term has genes and, in most cases, disease mutations associated. Therefore, panels can easily be defined from the viewpoint of the diseases. Selecting one or more diseases will include the corresponding genes and known diagnostic mutations to the definition of the panel. Then this selection can be customized. More mutations can be added in a graphical representation of the genomic environment. Also genes can be added o removed to adjust the panel to the real regions captured by targeted enrichment.

#### Defining panels with the panel manager

**Figure 1.**
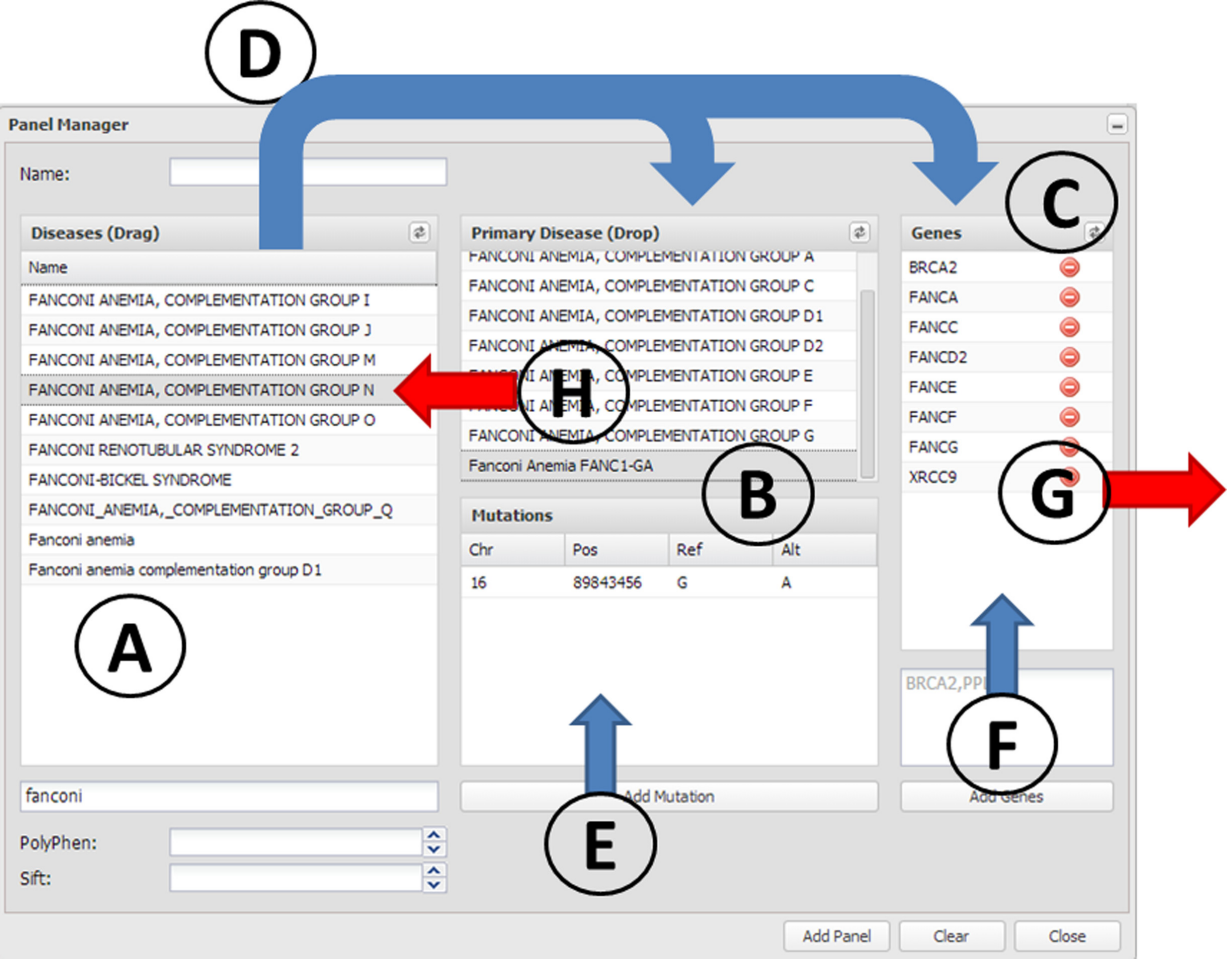
The panel manager. The elements used to define a panel are (**A**) disease terms, (**B**) diagnostic mutations and (**C**) genes. Arrows represent actions that can be taken in the panel manager. Panels can be defined by using the known mutations and genes of a particular disease. This can be done by dragging them to the ***Primary Diagnostic*** box (action **D**). This action, in addition to defining the diseases in the ***Primary Diagnostic*** box, automatically adds the corresponding genes to the ***Genes*** box. The panels can be customized by adding new genes (action **F**) or removing undesired genes (action **G**). New disease mutations can be added independently or associated to an already existing disease term (action **E**). Disease terms can be removed by simply dragging them back (action **H**).

The ***New panel*** button of the edit icon of a panel invokes the ***Panel manager*** (Figure 1). There are three elements corresponding to the three concepts used to define a panel, from left to right: *Diseases* (Figure 1A), *mutations* (Figure 1B) and *genes* (Figure 1C). *Genes* define the physical region of the genome that will be analyzed by the program that often corresponds to coding genes but, in general, can make reference to any genomic region(s). *Mutations* are the particular variants that will be used for the primary diagnostic. And *diseases* are the definitions of the particular phenotypes associated with the variants (although not necessarily to coding genes).

The easiest way to start defining a panel is by using the known mutations and genes of a particular disease. To do so, the disease terms (Figure 1A) can be dragged and dropped (Figure 1D) on the ***Primary Diagnostic*** box (Figure 1B upper box). Currently, TEAM manages 10 095 disease terms obtained from the disease databases (HGMD-public, HUMSAVAR, ClinVar and COSMIC) by means of CellBase.

As previously indicated, each disease term has the corresponding disease genes associated through the above mentioned disease databases. Therefore, this action (Figure 1D), in addition to defining the diseases in the ***Primary Diagnostic*** box, automatically adds the corresponding genes to the ***Genes*** list (Figure 1C). The panels so defined are configurable: new genes can be added by filling in the box behind the Genes list and pressing the ***Add genes*** button (Figure 1F). Alternatively, undesired genes can be removed by pressing in the corresponding icon (Figure 1G). Additionally, the genomic regions included in the panel can be defined by their chromosomal coordinates in the widely used Browser Extensible Data (BED) format.

Disease terms can be removed by simply dragging them back (Figure 1H).

Any disease term has at least one disease mutation associated (all of them have been extracted from mutation databases). Currently, TEAM has information on 39 720 218 disease variants and mutations from HGMD-public, HUMSAVAR, ClinVar and COSMIC, obtained via CellBase. These disease mutations constitute the knowledge for the diagnostic. This is considered public domain information and cannot be edited. However, it might happen that new disease mutations are known but they are not included in the databases yet. In this case it is also possible to add new diagnostic mutations within an intuitive environment that helps to locate the variants in the proper position within the gene coordinates. By clicking the ***Add mutation*** button, the corresponding ***Add mutation*** window is invoked. This window provides an intuitive environment to locate mutated positions in the genomic coordinates. It includes an embedded genome viewer, the Genome Maps (13). By means of the search option the user can immediately be located over the gene of interest. The chromosome, position and reference allele are automatically updated as the user moves along the genome. Alternatively, a chromosome number and a genomic location can be provided and pressing the ***Check*** button focuses on the chosen coordinates. Once the position of the mutation is located, the alternative allele must be specified. Finally, by clicking the ***Add Mutation*** button, the mutation is added. If the disease name corresponds to any disease term, then the mutation will be added to it.

Additionally, new mutations can be added to any disease term in the ***Primary Disease*** box (Figure 1B upper box) by clicking on it with the right mouse button and choosing the ***Add mutation*** option. The added mutations are displayed in the ***Mutation*** box when the corresponding disease term is selected.

### TEAM technical features

TEAM is an open source tool based on HTML5 standard. The application front-end is developed in Javascript using the Ext JS and Boostrap frameworks. The entire application runs locally. The system uses HTML5 local storage and no data is sent to the server. Only the information on genes and disease variants corresponding to panel definitions are obtained from the web services of CellBase (14) (http://wiki.opencb.org/projects/cloud/doku.php?id=cellbase:overview), which includes data from different disease-related variant databases (HGMD-public, HUMSAVAR, ClinVar and COSMIC).

## DISCUSSION

A recognized drawback for many applications derived from high throughput sequencing technologies is the lack of software tools to properly deal with the enormous amounts of data they generate and to relate these data to the vast amount of biological knowledge available. In particular, disease-targeted sequencing is becoming popular in clinic because it enables comprehensive and cost-effective diagnostic. However, to our knowledge there are no specific tools to deal with disease-targeted sequencing data. The tool described here, TEAM, provides an intuitive environment for the clinician in which unprocessed data on patient's genomic variation can easily be transformed in a diagnostic. Moreover, when this initial diagnostic is not possible with the current knowledge on disease-related variants, a list of secondary findings that can eventually result in a successful diagnostic is provided. Probably, the best feature of TEAM is its interactive interface for the definition of and customization of panels. By means of this interface, genes and mutations can be added or discarded to adjust panel definitions. Alternatively, this customization can be used to apply virtual panels to whole exome or genome sequencing experiments to reduce the number of secondary findings. Virtual panels are flexible and can be immediately changed and the knowledge on the disease evolves.

From a technical point of view, TEAM represents a new philosophy in which remote highly efficient database query systems move the information to the local genomic data rather than the opposite, as many other programs do. An advantage derived from this is that patient's data are locally managed and therefore potential confidentiality issues are avoided.
